# Store‐operated interactions between plasmalemmal STIM1 and TRPC1 proteins stimulate PLCβ1 to induce TRPC1 channel activation in vascular smooth muscle cells

**DOI:** 10.1113/JP273302

**Published:** 2016-12-07

**Authors:** Jian Shi, Francesc Miralles, Lutz Birnbaumer, William A. Large, Anthony P. Albert

**Affiliations:** ^1^ Vascular Biology Research CentreMolecular & Clinical Sciences Research Institute; ^2^ Institute of Medical & Biomedical EducationSt George'sUniversity of LondonLondonUK; ^3^ Neurobiology LaboratoryNational Institute of Environmental Health SciencesResearch Triangle ParkNCUSA; ^4^ Institute of Biomedical Research (BIOMED)School of Medical SciencesCatholic University of ArgentinaBuenos AiresArgentina

**Keywords:** PLC, STIM1, TRPC, vascular smooth muscle

## Abstract

**Key points:**

- Depletion of Ca^2+^ stores activates store‐operated channels (SOCs), which mediate Ca^2+^ entry pathways that regulate cellular processes such as contraction, proliferation and gene expression.
- In vascular smooth muscle cells (VSMCs), stimulation of SOCs composed of canonical transient receptor potential channel 1 (TRPC1) proteins requires G protein α q subunit (Gαq)/phospholipase C (PLC)β1/protein kinase C (PKC) activity. We studied the role of stromal interaction molecule 1 (STIM1) in coupling store depletion to this activation pathway using patch clamp recording, GFP‐PLCδ1‐PH imaging and co‐localization techniques.
- Store‐operated TRPC1 channel and PLCβ1 activities were inhibited by STIM1 short hairpin RNA (shRNA) and absent in TRPC1^−/−^ cells, and store‐operated PKC phosphorylation of TRPC1 was inhibited by STIM1 shRNA. Store depletion induced interactions between STIM1 and TRPC1, Gαq and PLCβ1, which required STIM1 and TRPC1. Similar effects were produced with noradrenaline.
- These findings identify a new activation mechanism of TRPC1‐based SOCs in VSMCs, and a novel role for STIM1, where store‐operated STIM1‐TRPC1 interactions stimulate Gαq/PLCβ1/PKC activity to induce channel gating.

**Abstract:**

In vascular smooth muscle cells (VSMCs), stimulation of canonical transient receptor potential channel 1 (TRPC1) protein‐based store‐operated channels (SOCs) mediates Ca^2+^ entry pathways that regulate contractility, proliferation and migration. It is therefore important to understand how these channels are activated. Studies have shown that stimulation of TRPC1‐based SOCs requires G protein α q subunit (Gαq)/phospholipase C (PLC)β1 activities and protein kinase C (PKC) phosphorylation, although it is unclear how store depletion stimulates this gating pathway. The present study examines this issue by focusing on the role of stromal interaction molecule 1 (STIM1), an endo/sarcoplasmic reticulum Ca^2+^ sensor. Store‐operated TRPC1 channel activity was inhibited by TRPC1 and STIM1 antibodies and STIM1 short hairpin RNA (shRNA) in wild‐type VSMCs, and was absent in TRPC1^−/−^ VSMCs. Store‐operated PKC phosphorylation of TRPC1 was reduced by knockdown of STIM1. Moreover, store‐operated PLCβ1 activity measured with the fluorescent phosphatidylinositol 4,5‐bisphosphate/inositol 1,4,5‐trisphosphate biosensor GFP‐PLCδ1‐PH was reduced by STIM1 shRNA and absent in TRPC1^−/−^ cells. Immunocytochemistry, co‐immunoprecipitation and proximity ligation assays revealed that store depletion activated STIM1 translocation from within the cell to the plasma membrane (PM) where it formed STIM1‐TRPC1 complexes, which then associated with Gαq and PLCβ1. Noradrenaline also evoked TRPC1 channel activity and associations between TRPC1, STIM1, Gαq and PLCβ1, which were inhibited by STIM1 knockdown. Effects of N‐terminal and C‐terminal STIM1 antibodies on TRPC1‐based SOCs and STIM1 staining suggest that channel activation may involve insertion of STIM1 into the PM. The findings of the present study identify a new activation mechanism of TRPC1‐based SOCs in VSMCs, and a novel role for STIM1, in which store‐operated STIM1‐TRPC1 interactions stimulate PLCβ1 activity to induce PKC phosphorylation of TRPC1 and channel gating.

## Key points

- Depletion of Ca^2+^ stores activates store‐operated channels (SOCs), which mediate Ca^2+^ entry pathways that regulate cellular processes such as contraction, proliferation and gene expression.
- In vascular smooth muscle cells (VSMCs), stimulation of SOCs composed of canonical transient receptor potential channel 1 (TRPC1) proteins requires G protein α q subunit (Gαq)/phospholipase C (PLC)β1/protein kinase C (PKC) activity. We studied the role of stromal interaction molecule 1 (STIM1) in coupling store depletion to this activation pathway using patch clamp recording, GFP‐PLCδ1‐PH imaging and co‐localization techniques.
- Store‐operated TRPC1 channel and PLCβ1 activities were inhibited by STIM1 short hairpin RNA (shRNA) and absent in TRPC1^−/−^ cells, and store‐operated PKC phosphorylation of TRPC1 was inhibited by STIM1 shRNA. Store depletion induced interactions between STIM1 and TRPC1, Gαq and PLCβ1, which required STIM1 and TRPC1. Similar effects were produced with noradrenaline.
- These findings identify a new activation mechanism of TRPC1‐based SOCs in VSMCs, and a novel role for STIM1, where store‐operated STIM1‐TRPC1 interactions stimulate Gαq/PLCβ1/PKC activity to induce channel gating.

**Abbreviations**
BAPTA: 1,2‐bis‐(2‐aminophenoxy)ethane‐*N*,*N*,*N*′,*N*′‐tetraacetic acid(acetoxymethyl ester)
BAPTA‐AM: 1,2‐bis(2‐aminophenoxy)ethane‐*N*,*N*,*N*′,*N*′‐tetraacetic acid acetoxymethyl ester
CPA: cyclopiazonic acid
CRACs: calcium release activated channels
ER/SR: endoplasmic/sarcoplasmic reticulum
Gαq: G protein α q subunit
InsP_3_: inositol 1,4,5‐trisphosphate
NP_o_: open probability
PIP_2_: phosphatidylinositol 4,5‐bisphosphate
PKC: protein kinase C
PLA: proximity ligation assay
PLC: phospholipase C
PM: plasma membrane
shRNA: short hairpin RNA
SOCs: store‐operated channels
STIM1: stromal interaction molecule 1
TPEN: *N*,*N*,*N*′,*N*′‐tetrakis(2‐pyridylmethyl)ethane‐1,2‐diamineed
TRPC: transient receptor potential channel
VSMCs: vascular smooth muscle cells
WT: wild‐type

## Introduction

Ca^2+^‐permeable store‐operated channels (SOCs) are physiologically activated by stimulation of the classical phosphoinositol signalling pathway involving G protein α q subunit (Gαq)‐coupled receptors, phospholipase C (PLC) activation, phosphatidylinositol 4,5‐bisphosphate (PIP_2_) hydrolysis, inositol 1,4,5‐trisphosphate (InsP_3_) generation and InsP_3_‐mediated depletion of endo/sarcoplasmic reticulum (ER/SR) Ca^2+^ stores. In vascular smooth muscle cells (VSMCs), SOCs mediate Ca^2+^ entry pathways that regulate cellular functions such as contractility, proliferation and migration, and are potential therapeutic targets for cardiovascular diseases such as hypertension and atherosclerosis (Abramowitz & Birnbaumer, 2009; Beech, 2013; Earley & Brayden, 2015). Identifying the molecules involved in composing and activating SOCs is an important objective in vascular biology.

There is increasing evidence that cells express two distinct types of SOCs (Cheng *et al*. 2013). Prototypical SOCs, termed calcium release‐activated channels (CRACs) responsible for the calcium release activated current (*I*_crac_), have several characteristic properties, such as high Ca^2+^ permeability, pronounced inward rectification and unitary conductances in the fS range, and are composed of pore‐forming Orai1 proteins (Prakriya & Lewis, 2015). Orai1‐based CRACs are gated by store depletion through stromal interaction molecule 1 (STIM1), a Ca^2+^ sensor within ER/SR stores that undergoes oligomerization after store depletion and approaches the cytosolic surface of the plasma membrane (PM) where it interacts directly with Orai1 to induce channel opening (Prakriya & Lewis, 2015; Soboloff *et al*. 2012). It is also apparent that many cell types express SOCs that have very different properties to those of Orai1‐based CRACs, such as much lower Ca^2+^ permeability, relatively linear rectification and considerably larger unitary conductances, and they are proposed to be composed by the canonical transient receptor potential family of Ca^2+^‐permeable non‐selective cation channels (TRPC1–C7) (Cheng *et al*. 2013). It is relatively well‐accepted that TRPC1 are regulated by store depletion, although there is less evidence for the other channel subtypes. TRPC‐based SOCs probably form a diverse family of structures as TRPC subunits form heteromeric channels (Alfonso *et al*. 2008; Saleh *et al*. 2008; Shi *et al*. 2012; Cheng *et al*. 2013). Whether TRPC proteins form a distinct family of SOCs remains controversial because it is unclear how store depletion couples to channel activation. However, there is growing support for STIM1 also being involved in the activation of TRPC‐based SOCs through both Orai1‐dependent and independent mechanisms (Ambudkar *et al*. 2007; Worley *et al*. 2007; Yuan *et al*. 2009; Cheng *et al*. 2011; Cheng *et al*. 2013; Liao *et al*. 2014).

It is proposed that VSMCs differentially express Orai1‐based CRACs and/or TRPC‐based SOCs according to their phenotype. In cells expressing a contractile phenotype, such as acutely isolated VSMCs and primary cultured VSMCs maintained under low serum conditions, SOCs have relatively linear rectification, unitary conductances of ∼2 pS and are composed of a heteromeric TRPC1/C5 molecular template that may also contain other TRPC subunits (Saleh *et al*. 2008; Albert *et al*. 2009; Large *et al*. 2009; Albert 2011; Shi *et al*. 2012). Because TRPC1 is essential for conferring gating by store depletion, these channels are termed TRPC1‐based SOCs (Shi *et al*. 2012). Importantly, SOCs with properties similar to Orai1‐based CRACs have not been described in contractile VSMCs (Shi *et al*. 2012), and Orai proteins are poorly expressed in these cells (Berra‐Romani *et al*. 2008; Trebak, 2012). In contrast, long‐term cultured VSMCs maintained under high serum conditions, which display a non‐contractile, synthetic phenotype associated with cell proliferation and migration, express both TRPC1‐based SOCs (Li *et al*. 2008; Ng et al., 2009, 2010) and Orai1‐based CRACs (Potier *et al*. 2009; Li *et al*. 2011; Beech, 2012; Trebak, 2012). In the present study, we examined the activation mechanisms of native TRPC1‐based SOCs in contractile VSMCs that probably mediate Ca^2+^ entry pathways involved in regulating vascular tone and the switching of VSMCs from contractile to synthetic phenotypes (Matchkov *et al*. 2013).

Our previous findings have revealed that protein kinase C (PKC) activity is pivotal for stimulation of TRPC1‐based SOCs in contractile VSMCs (Saleh *et al*. 2008; Albert *et al*. 2009; Large *et al*. 2009; Albert 2011; Shi *et al*. 2012). It is proposed that store‐operated PKC phosphorylation of TRPC1 is necessary for PIP_2_ binding to occur, which acts as the gating ligand (Saleh *et al*. 2009; Albert *et al*. 2009; Large *et al*. 2009; Albert 2011; Shi et al., 2012, 2014). This process is controlled by the PIP_2_‐binding protein myristoylated alanine‐rich C kinase substrate, which behaves as a reversible PIP_2_ buffer to control the local PIP_2_ levels required for channel activation (Shi *et al*. 2014). In a recent study, we investigated how depletion of stores induces this activation pathway and revealed that store depletion stimulates Gαq‐evoked PLCβ1 activity to induce PKC phosphorylation of TRPC1 proteins (Shi *et al*. 2016). However, these studies did not determine how store depletion is coupled to Gαq/PLCβ1 activity. The present study investigates this question, and identifies a new activation mechanism of TRPC1‐based SOCs in VSMCs, as well as a novel role for STIM1, in which store depletion induces the formation of STIM1‐TRPC1 complexes that stimulate PLC activity.

## Methods

### Cell isolation

New Zealand White rabbits (2–3 kg) were killed using i.v. sodium pentobarbitone (120 mg kg^−1^) and wild‐type (WT) and TRPC1^−/−^ mice were killed using cervical dislocation according to UK Animals Scientific Procedures Act of 1986 and as revised by European Directive 2010/63/EU. All experiments were carried out in accordance with guidelines laid down by St George's, University of London Animal Welfare Committee, and conform with the principles and regulations described by Grundy (2015). Portal veins from rabbit or second‐order mesenteric arteries from mice were dissected free, and then cleaned of fat, connective tissue and endothelium in physiological salt solution containing (mm): 126 NaCl, 6 KCl, 10 glucose, 11 Hepes, 1.2 MgCl_2_ and 1.5 CaCl_2_ (pH adjusted to 7.2 using 10 m NaOH). Vessels were enzymatically dispersed into single VSMCs as described previously (Shi *et al*. 2016).

### Primary cell culture

VSMCs were seeded into culture plates; maintained using Dulbecco's modified Eagle's medium/F‐12 media containing 1% serum, and incubated at 37°C in 95%O_2_:5%CO_2_ at 100% humidity for up to 7 days. In 1% serum, VSMCs maintained their contractile phenotype and had properties similar to TRPC1 channel currents in freshly dispersed VSMCs (Shi *et al*. 2016), suggesting that compensatory changes to channel properties probably did not take place under these cell culture conditions.

### Electrophysiology

Whole‐cell and single‐channel cation currents were recorded with an AXOpatch 200B amplifier (Axon Instruments, Union City, CA, USA) at room temperature (20–23°C) as described previously (Shi *et al*. 2016). Whole‐cell currents were filtered at 1 kHz (−3 dB, low‐pass 8‐pole Bessel filter, Frequency Devices model LP02; Scensys, Aylesbury, UK) and sampled at 5 kHz (Digidata 1322A and pCLAMP, version 9.; Molecular Devices, Sunnydale, CA, USA). Whole‐cell current/voltage (I/V) relationships were obtained by applying 750 ms duration voltage ramps from +100 to −150 mV every 30 s from a holding potential of 0 mV. Single‐channel currents were filtered between 0.1 and 0.5 kHz and acquired at 1–5 kHz. Single‐channel *I*/*V* relationships were evaluated by manually altering the holding potential of −80 mV between −120 and +120 mV. Single‐channel current amplitudes were calculated from idealized traces of ≥60 s in duration using the 50% threshold method and analysed using pCLAMP, version 9.0. Events lasting for < 6.664 ms (2 × rise time for a 100 Hz (−3 dB) low‐pass filter) were excluded from analysis to maximize the number of channel openings reaching their full current amplitude. Open probability (NP_O_) was used as a measure of channel activity and was calculated automatically using pCLAMP, version 9. Single‐channel current amplitude histograms were plotted from the event data of the idealized traces with a bin width of 0.01 pA. Amplitude histograms were fitted using Gaussian curves with peak values corresponding to channel open levels. Mean channel amplitudes at different membrane potentials were plotted, and *I*/*V* relationships were fitted by linear regression with the gradient determining conductance values. Images were prepared using Origin, version 6.0 (MicroCal Software, Northampton, MA, USA), in which inward single‐channel openings are shown as downward deflections.

Whole‐cell recording bath solution contained (mm): 126 NaCl, 1.5 CaCl_2_, 10 Hepes, 11 glucose, 0.1 4,4‐diisothiocyanostilbene‐2,2‐disulphonic acid, 0.1 niflumic acid, and 0.005 nicardipine, pH to 7.2 with NaOH. Under these conditions, voltage‐dependent Ca^2+^ channels and Ca^2+^‐activated and swell‐activated Cl^−^ conductances are blocked, allowing cation conductances to be recorded in isolation. Whole‐cell patch pipette and inside‐out patch bathing solutions contained (mm): 18 CsCl, 108 cesium aspartate, 1.2 MgCl_2_, 10 Hepes, 11 glucose, 1 Na_2_ATP and 0.2 NaGTP (pH adjusted to 7.2 with Tris). Free [Ca^2+^]_i_ was set at 100 nm by adding 4.8 mm CaCl_2_ plus 10 mm 1,2‐bis‐(2‐aminophenoxy)ethane‐*N*,*N*,*N*′,*N*′‐tetraacetic acid(acetoxymethyl ester) (BAPTA) or 0.48 mm CaCl_2_ plus 1 mm BAPTA for whole‐cell and inside‐out recordings respectively using EqCal (Biosoft, Cambridge, UK). In cell‐attached patch experiments, the membrane potential was set to 0 mV by perfusing cells in a KCl external solution containing (mm): 126 KCl, 1.5 CaCl_2_, 10 Hepes and 11 glucose (pH adjusted to 7.2 with 10 m KOH). Nicardipine (5 μm) was included to prevent smooth muscle cell contraction by blocking Ca^2+^ entry through voltage‐dependent Ca^2+^ channels. The patch pipette solution used for both cell‐attached and inside‐out patch recording (extracellular solution) was K^+^ free and contained (mm): 126 NaCl, 1.5 CaCl_2_, 10 Hepes, 11 glucose, 10 TEA, 5 4‐AP, 0.0002 iberiotoxin, 0.1 4,4‐diisothiocyanostilbene‐2,2‐disulphonic acid, 0.1 niflumic acid and 0.005 nicardipine (pH adjusted to 7.2 with NaOH).

### Knockdown of STIM1 and PLCβ1

We used lentiviral‐mediated delivery of pLKO.1‐puro based short hairpin RNA (shRNA) expression plasmids to knockdown STIM1 and PLCβ1 (Sigma‐Aldrich, Gillingham, UK). Infected VSMCs were selected with 2.5 μg ml^−1^ puromycin (Invitrogen, San Diego, CA, USA) for 2 days prior to the experiments, and STIM1 and PLCβ1 levels were determined by western blotting. Two STIM1 shRNA were used to knockdown proteins in rabbits (Sequence 1: 5′‐CACCTTCCATGGTGAGGATAA‐3′ and Sequence 2: 5′‐GGCTGCTGGTTTGCCTATATC‐3′) and two different sequences were used to knockdown STIM1 in mice (Sequence 1: 5′‐CACCTTCCATGGTGAGGATAA‐3′ and Sequence 2: 5′‐CCCTTCCTTTCTTTGCAATAT‐3′). PLCβ1 shRNA1 (5′‐GCAGATAAACATGGGCATGTA‐3′) and shRNA2 (5′‐GCTGTCTTTGTCTACATAGAA‐3) were used to knockdown PLCβ1 in both rabbits and mice (Shi *et al*. 2016). Scrambled shRNA sequences from STIM1 shRNA1/2 and PLCβ1 shRNA1/2 were used as controls.

### Imaging of GFP‐PLCδ‐PH‐mediated signals

VSMCs were transfected with GFP‐PLCδ‐PH (plasmid ID:21179; Addgene, Cambridge, MA, USA) using Nucleofector in accordance with the manufacturer's instructions (Amaxa Biosystems, Gaithersburg, MD, USA). Next, 0.2–0.4 μg of plasmid DNA was added to 1 × 10^5^ cells re‐suspended in 20 μl of Nucleofector solution, and cells were kept in primary cell culture conditions for up to 3 days. Transfected cells were imaged using an LSM 510 laser scanning confocal microscope and associated software (Carl Zeiss, Jena, Germany). Excitation was produced by 488/405 nm lasers and delivered via an Apochromat 63× oil‐immersion objective (numerical aperture, 1.4) (Carl Zeiss). Two‐dimensional images cut horizontally through approximately the middle of the cells were captured (1024 × 1024 pixels). Final images were produced using PowerPoint (Microsoft Inc., Redmond, WA, USA).

### Immunoprecipitation and western blotting

Freshly isolated vessel segments or primary cultured cells were lysed by sonication for 3 h in RIPA buffer (Santa Cruz Biotechnology, Santa Cruz, CA, USA) and then transferred to a microcentrifuge tube and cleared by centrifuging at 1000 *g* for 10 min at 4°C. Total cell lysate protein was immunoprecipitated using antibodies raised against targeted proteins with a Millipore Catch and Release Kit (Millipore, Billerica, MA, USA) followed by one‐dimensional protein gel electrophoresis (15–20 μg of total protein/lane). Separated proteins were transferred onto polyvinylidene difluoride membranes, and then membranes were incubated with 5% (weight/volume) non‐fat milk in PBS containing 0.1% Tween 20 to block non‐specific binding, and then primary antibodies were added and the membrane left overnight at 4°C. Visualization was performed with a horseradish peroxidase‐conjugated secondary antibody (80 ng ml^−1^) and ECL chemiluminescence reagents (Pierce Biotechnology, Inc., Rockford, IL, USA) for 1 min and exposure to photographic films. Band intensities were calculated using Image Studio software (Li‐Cor Biosciences Ltd, Cambridge, UK) and then were normalized to control bands. Data shown represent the findings obtained from ≥3 different animals.

### Immunocytochemistry

Freshly isolated VSMCs were fixed with 4% paraformaldehyde (Sigma‐Aldrich) for 10 min, washed with PBS, and permeabilized with PBS containing 0.1% Triton X‐100 for 20 min at room temperature. Cells were incubated with PBS containing 1% BSA for 1 h at room temperature and then were incubated with primary antibodies in PBS containing 1% BSA overnight at 4°C. In control experiments, cells were incubated without the primary antibody. The cells were washed and incubated with secondary antibodies conjugated to a fluorescence probe. Unbound secondary antibodies were removed by washing with PBS, and nuclei were labelled with 4′,6‐diamidino‐2‐phenylindole (DAPI) mounting medium (Sigma‐Aldrich). Cells were imaged using an LSM 510 laser scanning confocal microscope (Carl Zeiss). The excitation beam was produced by an argon (488 nm) or helium/neon laser (543 nm and 633 nm) and delivered to the specimen via an Apochromat 63× oil‐immersion objective (numerical aperture, 1.4) (Carl Zeiss). Emitted fluorescence was captured using LSM 510 software (release 3.2; Carl Zeiss). Two‐dimensional images cut horizontally through approximately the middle of the cells were captured (1024 × 1024 pixels). Raw confocal imaging data were processed and analysed using LSM 510 software. Final images were produced using PowerPoint (Microsoft Inc.).

### Proximity ligation assay

Freshly isolated VSMCs were studied using the Duolink *in situ* proximity ligation assay (PLA) detection kit 563 (Olink, Uppsala, Sweden) (Soderberg *et al*. 2008). Cells were adhered to coverslips, fixed in PBS containing 4% paraformaldehyde for 15 min, and permeabilized in PBS containing 0.1% Triton X‐100 for 15 min. Cells were blocked for 1 h at 37°C in blocking solution, and incubated overnight at 4°C with anti‐STIM1 and anti‐TRPC1 antibodies (both at dilution 1:200) in antibody diluent solution. Cells were labelled with combinations of either anti‐goat PLUS/anti‐rabbit MINUS or anti‐goat PLUS/anti‐mouse MINUS depending on animal species used for 1 h at 37°C. Hybridized oligonucleotides were ligated for 30 min at 37°C prior to amplification for 100 min at 37°C. Red fluorescence‐labelled oligonucleotides were then hybridized to rolling circle amplification products, and visualized using a Confocal LSM 510 (Carl Zeiss).

### Reagents

Rabbit anti‐TRPC1 antibody was generated by GenScript (Piscataway, NJ, USA) using peptide sequences from a previously characterized putative extracellular region (Xu & Beech, 2001). Goat anti‐TRPC1 (sc‐15055), mouse anti‐STIM1 (sc‐393705), goat anti‐STIM1 (sc‐79106), goat anti‐TRPC6 (sc‐19196), mouse anti‐Gαq (sc‐136181), goat anti‐PLCβ1 (sc‐31755) and mouse anti‐PLCβ1 (sc‐5291) antibodies were obtained from Santa Cruz Biotechnology (Dallas, TX, USA). All secondary antibodies were obtained from Santa Cruz Biotechnology. Alexa fluor 488‐conjugated donkey anti‐rabbit antibodies and Fluor 546‐conjugated donkey anti‐mouse antibodies were obtained from Thermo Fisher Scientific (Waltham, MA, USA). Mouse anti‐β‐actin antibody (A1978) was obtained from Sigma‐Aldrich. Rabbit anti‐STIM1 antibody against the N‐terminal EF hand (11565‐1‐AP) was obtained from Proteintech (Chicago, IL, USA), mouse anti‐GOK/STIM1 (610954) against the N‐terminal EF hand was obtained from BD Biosciences (Oxford, UK) and mouse anti‐STIM1 antibody against the C‐terminal region (SC‐66173) was obtained from Santa Cruz. All other drugs were purchased from Sigma‐Aldrich, or Tocris (Abingdon, UK). Agents were dissolved in distilled H_2_O or 0.1% DMSO. DMSO alone had no effect on whole‐cell currents or single‐channel activity.

### Statistical analysis

Statistical analysis was performed using paired (comparing the effects of agents on the same cell) or unpaired (comparing the effects of agents between cells) Student's *t* tests. *P *< 0.05 was considered statistically significant.

## Results

### TRPC1 compose SOCs in contractile VSMCs

**Figure 1:**
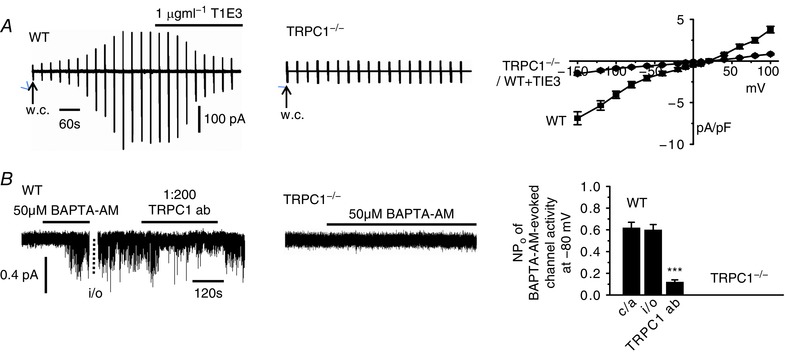
TRPC1 compose SOCs in contractile VSMCs *A*, development of a store‐operated whole‐cell current in a mesenteric artery VSMC from a WT mouse following break‐in into the whole‐cell configuration (w.c.) was inhibited by bath application of T1E3. Vertical deflections represent currents evoked by voltage ramps from +100 mV to −150 mV (750 ms duration) every 30 s from a holding potential of 0 mV. Development of a store‐operated whole‐cell TRPC1 current was absent in a TRPC1^−/−^ VMSC. Mean *I*/*V* relationships of store‐operated whole‐cell currents demonstrate that T1E3 reduced store‐operated whole‐cell currents at all membrane potentials tested in WT VSMCs and that store‐operated currents were absent in TRPC1^−/−^ VSMCs (each point is at least *n* = 6). *B*, BAPTA‐AM evoked single channel activity in a cell‐attached patch (c/a) held at −80 mV from a WT VSMC was maintained following patch excision into the inside‐out configuration (*i*/*o*). Bath application of an intracellular acting anti‐TRPC1 antibody to the cytosolic surface of the inside‐out patch inhibited BAPTA‐evoked channel activity. BAPTA‐AM failed to activate channel activity in a cell‐attached held at −80 mV from a TRPC1^−/−^ VSMC. Mean data of the inhibitory effect of the anti‐TRPC1 antibody on BAPTA‐evoked channel activity. Note that channel activities were maintained on changing from cell‐attached to inside‐out patch configurations (*n* = 7; ^***^ *P *< 0.001).

In our initial experiments, we confirmed that native TRPC1‐based SOCs are functionally expressed in contractile VSMCs using freshly isolated mesenteric artery VSMCs from WT and TRPC1^−/−^ mice, and antibodies raised against TRPC1 as channel blockers (Xu & Beech, 2001; Xu *et al*. 2005; Saleh *et al*. 2008; Albert *et al*. 2009; Large *et al*. 2009; Albert, 2011; Shi *et al*. 2012; Shi *et al*. 2016). In WT VSMCs, passive depletion of internal Ca^2+^ stores following cell dialysis with a patch pipette solution containing high concentrations of BAPTA and no added Ca^2+^ evoked whole‐cell cation currents with a relatively linear current‐voltage (*I*/*V*) relationship and an *E*_rev_ of +20 mV, which were inhibited by bath application of TIE3, a TRPC1 antibody raised against a putative extracellular channel pore site (Xu & Beech, 2001; Xu *et al*. 2005), by >80% at all membrane potentials tested (Fig. 1 *A*). In WT VSMCs, bath application of the cell‐permeable Ca^2+^ chelator 1,2‐bis(2‐aminophenoxy)ethane‐*N,N,N′,N′*‐tetraacetic acid acetoxymethyl ester (BAPTA‐AM), also activated single‐channel activity in cell‐attached patches with a unitary conductance of ∼2 pS, which was inhibited by bath application of an TRPC1 antibody raised against an putative intracellular site by ∼85% at −80 mV following patch excision into the inside‐out configuration (Fig. 1 *B*). These whole‐cell and single‐channel current properties are similar to those of native TRPC1‐based SOCs previously described in VSMCs from various vascular beds and different animal species (Saleh *et al*. 2008; Albert *et al*. 2009; Large *et al*. 2009; Albert, 2011; Shi *et al*. 2012; Shi *et al*. 2016). In further agreement with earlier findings, store‐operated whole‐cell conductances and single‐channel activities were absent in TRPC1^−/−^ VSMCs (Shi *et al*. 2012) (Fig. 1). These results clearly indicate that native TRPC1‐based SOCs are functionally expressed in contractile VSMCs.

### STIM1 is obligatory for activation of TRPC1 SOCs

**Figure 2:**
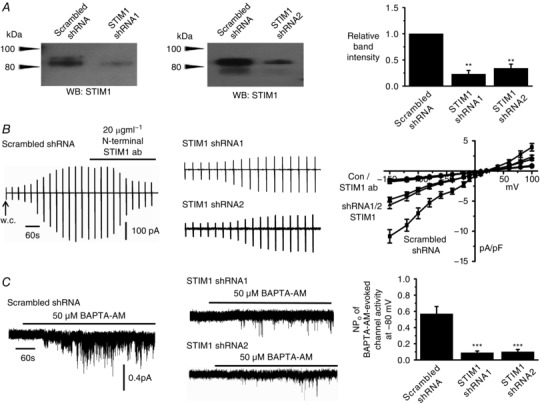
TRPC1‐based SOCs are dependent on STIM1 *A*, western blots and mean data confirm that two different STIM1 shRNA sequences (shRNA1 and shRNA2) reduced STIM1 expression (three primary rabbit portal vein VSMC culture preparations; ^**^ *P* < 0.01). *B*, traces and mean *I*/*V* relationships showing that peak amplitude of store‐operated whole‐cell TRPC1‐based currents were greatly reduced at all membrane potentials tested following transduction of rabbit portal vein VSMCs with shRNA sequences compared to scrambled shRNA sequences. In the presence of scrambled sequences, store‐operated TRPC1‐based currents were inhibited by an anti‐STIM1 antibody (*n* = 6). *C*, recordings and mean data showing that BAPTA‐AM‐evoked TRPC1‐based SOCs were reduced by shRNA sequences targeting STIM1 compared to scrambled shRNA in VSMCs (*n* = 6; ^***^ *P* < 0.001).

We next examined whether STIM1 is involved in activating TRPC1‐based SOCs in contractile VSMCs using a knockdown strategy, and an antibody raised against the N‐terminal EF hand of STIM1 (Spassova *et al*. 2006; Hewavitharana *et al*. 2008). Western blot studies showed that STIM1 protein is expressed in primary cultured rabbit portal vein VSMCs, maintained under low concentrations of fetal calf serum to retain their contractile phenotype (Shi *et al*. 2016), and that two different STIM1 shRNA sequences reduced STIM1 expression by ∼80% compared to scrambled shRNA sequences (Fig. 2 *A*). In control experiments, STIM1 shRNA1 did not alter TRPC1, Gαq, PLCβ1 and β‐actin expression levels (data not shown).

In VSMCs expressing scrambled shRNA, development of store‐operated whole‐cell TRPC1 currents remained unaffected, although peak currents were inhibited by >80% at all membrane potentials tested following bath application of the N‐terminal STIM1 antibody (Fig. 2 *B*). Treatment of cells with STIM1 shRNAs reduced development of store‐operated whole‐cell TRPC1 currents by about 60% at all membrane potentials tested (Fig. 2 *B*), and inhibited BAPTA‐AM‐evoked single TRPC1 channel activities by >80% at −80 mV (Fig. 2 *C*). In addition, the SR Ca^2+^‐ATPase inhibitor cyclopiazonic acid (CPA) and the cell‐permeable low affinity Ca^2+^ chelator *N*,*N*,*N*‘,*N*’‐tetrakis(2‐pyridylmethyl)ethane‐1,2‐diamineed (TPEN) also activated TRPC1 channel activities, which were reduced by STIM1 shRNA1 and 2 (data not shown). These results clearly indicate that STIM1 is essential for activation of native TRPC1‐based SOCs in contractile VSMCs.

### Store‐operated PKC phosphorylation of TRPC1 requires STIM1

**Figure 3:**
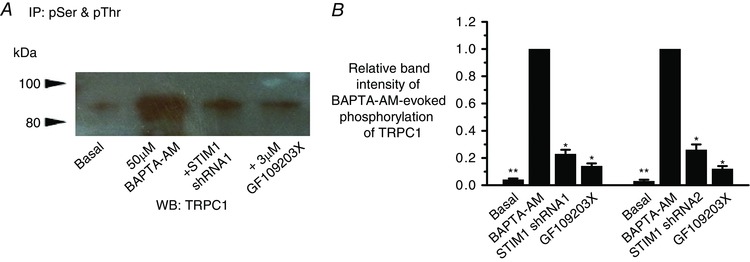
Store‐operated phosphorylation of TRPC1 proteins requires STIM1 *A*, co‐immunoprecipitation of rabbit portal vein tissue lysates with anti‐phosphorylated serine (pSer) and threonine (pThr) antibodies followed by western blotting (WB) with an anti‐TRPC1 antibody shows that basal TRPC1 phosphorylation is increased by pre‐treatment with BAPTA‐AM for 10 min, and that this increase was reduced by STIM1 shRNA1 and coapplication of GF109203X. *B*, mean relative band densities normalized to BAPTA‐AM bands (three different tissue lysate preparations; ^*^ *P *< 0.05, ^**^ *P *< 0.01). [Colour figure can be viewed at wileyonlinelibrary.com]

We have previously shown that PKC phosphorylation of TRPC1 is a pivotal event in the activation of TRPC1‐based SOCs in contractile VSMCs (Shi *et al*. 2016) and therefore we investigated whether STIM1 is involved in this gating pathway. Immunoprecipitation of isolated vessel lysates with a mixture of anti‐phosphorylated serine and threonine antibodies followed by western blotting with an anti‐TRPC1 antibody showed that TRPC1 proteins expressed low basal phosphorylation, which was greatly increased following pre‐treatment with BAPTA‐AM (Fig. 3). Expression of STIM1 shRNA1 and shRNA2 sequences and coapplication of the PKC inhibitor GF09203X greatly reduced BAPTA‐AM‐induced TRPC1 phosphorylation by >80% (Fig. 3). These findings clearly indicate that STIM1 is required for PKC phosphorylation of TRPC1 proteins by store depletion.

### Store depletion induces PLCβ1 activity mediated by STIM1 and TRPC1

In a recent study, we reported that store depletion stimulates Gαq‐evoked PLCβ1 activity, which drives PKC activity and gating of native TRPC1‐based SOCs in contractile VSMCs (Shi *et al*. 2016). However, it remains unclear how store depletion stimulates this pathway. The above findings reveal that STIM1 is central for store‐operated PKC phosphorylation of TRPC1 and channel activation and therefore we investigated whether this ER/SR Ca^2+^ sensor couples store depletion to stimulation of PLCβ1 activity. To monitor store‐operated PLCβ1 activity, we transfected primary cultured VSMCs with GFP‐PLCδ1‐PH, a fluorescence biosensor with a high affinity for PIP_2_ and InsP_3_ (Balla & Vamai, 2009; Szentpetery *et al*. 2009) and measured signal changes (measured as relative fluorescence units) at the PM (*F*_m_) and within the cytosol (*F*_c_) as described previously (Shi *et al*. 2016).

**Figure 4:**
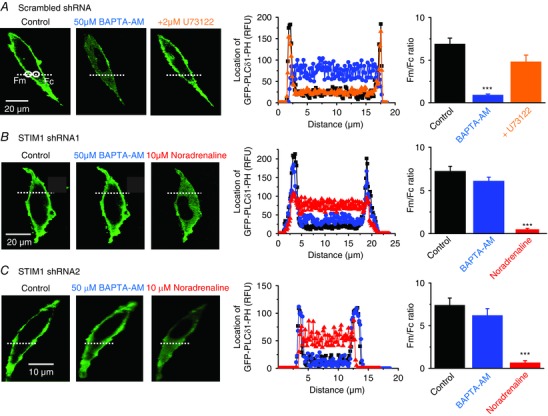
Store‐operated PLC activity is mediated by STIM1 *A*, image from a single rabbit portal vein VSMC showing that in control conditions the location of GFP‐PLCδ1‐PH‐mediated signals (measured as relative fluorescence units; RFU) was predominantly expressed at the PM (black). In the same cell, pre‐treatment with BAPTA‐AM for 10 min induced translocation of signals to the cytosol (blue), and coapplication of U73122 for 5 min reversed these cytosolic signals back to the PM (orange). Graphs of RFU of line scans for the region denoted along the white dotted lines show GFP‐PLCδ1‐PH signals across the cell width. Mean *F*_m_/*F*_c_ ratios of GFP‐PLCδ1‐PH‐mediated signals represent 20 cells from three different experiments (^***^ *P*<0.001). *B* and *C*, images and mean data showing that transduction of rabbit portal vein VSMCs with either STIM1 shRNA1 or shRNA2 sequences prevented BAPTA‐AM inducing translocation of GFP‐PLCδ1‐PH signals to the cytosol. Under both these conditions, application of noradrenaline for 5 min (red, applied in the presence of 1 μm Wortmannin to prevent cell contraction) was still able to induce translocation of GFP‐PLCδ1‐PH signals from the PM to the cytosol (20 cells for each STIM1 shRNA sequence from three different experiments; ^***^ *P *< 0.01).

In unstimulated VSMCs containing scrambled or STIM1 shRNAs, GFP‐PLCδ1‐PH signals had a mean *F*_m_/*F*_c_ ratio of ∼7, reflecting a predominant location of PIP_2_ at the PM (Fig. 4 *A*). In scrambled shRNA VSMCs, bath application of BAPTA‐AM produced a significant reduction in the mean *F*_m_/*F*_c_ ratio. This GFP‐PLCδ1‐PH signal change represents PLCβ1 activity stimulated by store depletion causing PIP_2_ hydrolysis at the PM and the subsequent generation of cytosolic InsP_3_ as described previously (Balla & Vamai, 2009; Szentpetery *et al*. 2009; Shi *et al*. 2016). In further agreement with our earlier study (Shi *et al*. 2016), BAPTA‐AM‐induced GFP‐PLCδ1‐PH signal changes were inhibited by coapplication the PLC inhibitor U73122 (Fig. 4 *A*). Similar effects on mean *F*_m_/*F*_c_ ratio were also produced with CPA and TPEN (data not shown).

Knockdown of STIM1 with shRNA1 and shRNA2 sequences greatly reduced translocation of GFP‐PLCδ1‐PH signals induced by BAPTA‐AM (Fig. 4 *B* and *C*) and CPA and TPEN (data not shown), indicating that STIM1 is the probable mediator that stimulates PLC activity when stores are depleted by BAPTA‐AM, CPA or TPEN.

Stimulation of endogenously expressed α1 Gαq‐coupled adrenoreceptors by bath application of noradrenaline induced translocation of GFP‐PLCδ1‐PH signals from the PM to the cytosol in the presence of STIM1 shRNA1 and 2 (Fig. 4 *B* and *C*). These results indicate that STIM1 independent pathways have a dominant role in stimulating PLC activity by this concentration of noradrenaline, and that knockout of STIM1 *per se* does not prevent activation of PLC. These findings do not exclude the possibility that noradrenaline‐stimulated STIM1 activity produces a small contribution to overall PLC activity, which is sufficient to activate TRPC1‐based SOCs (see Fig. 11 and accompanying text).

**Figure 5:**
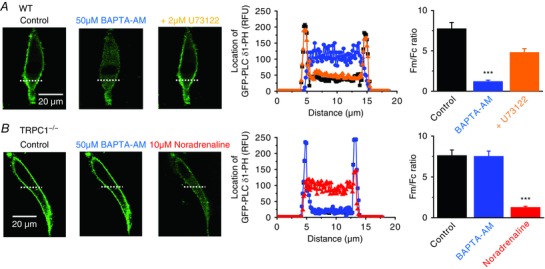
Store‐operated PLC activity requires TRPC1 *A*, WT mesenteric artery VSMCs: application of BAPTA‐AM for 10 min induced translocation of GFP‐PLCδ1‐PH‐mediated signals from the PM to the cytosol which was prevented by coapplication of U73122 for 5 min (20 cells from three different experiments; ^***^ *P *< 0.001). *B*, TRPC1^−/−^ VSMCs: BAPTA‐AM did not alter the cellular distribution of GFP‐PLCδ1‐PH signals whereas application of noradrenaline for 5 min induced translocation of GFP‐PLCδ1‐PH signals from the PM to the cytosol (20 cells from three different experiments; ^***^ *P *< 0.001).

Because our findings indicate that STIM1 is obligatory for activation of TRPC1‐based SOCs and store‐operated PLC activity but is not required for PLC activity *per se*, we investigated whether TRPC1 is also essential for coupling store depletion to stimulation of PLC activity. In VSMCs from WT mice, BAPTA‐AM evoked a translocation of GFP‐PLCδ1‐PH signals from the PM to the cytosol similar to those in rabbit VSMCs (Fig. 4 *A*) and these signal changes were also reduced by coapplication of U73122 (Fig. 5 *A*). By contrast, BAPTA‐AM failed to alter the cellular distribution of GFP‐PLCδ1‐PH signals in TRPC1^−/−^ VSMCs, although noradrenaline was capable of evoking substantial translocation of GFP‐PLCδ1‐PH signals (Fig. 5 *B*). Similar effects between WT and TRPC1^−/−^ mice were produced with CPA and TPEN (data not shown). These data suggest that TRPC1, similar to STIM1, is an important determinant for store‐operated PLC activity but it is not an absolute requirement for Gαq‐coupled receptor‐stimulated PLC activity.

### Store depletion induces interactions between STIM1, TRPC1, Gαq and PLCβ1

The present study proposes that both STIM1 and TRPC1 are required for store depletion to stimulate PLCβ1 activity, and therefore we hypothesized that store depletion probably induces associations between STIM1 and TRPC1 at (or near) the PM.

**Figure 6:**
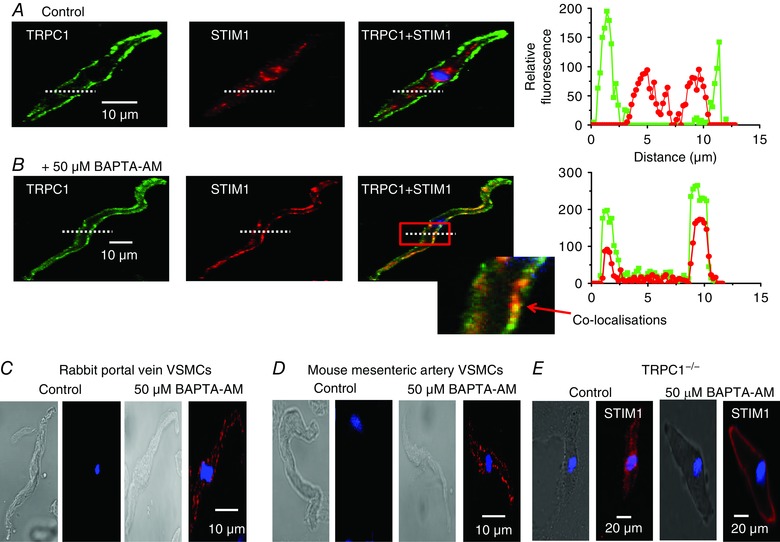
Store‐depletion induces co‐localizations between STIM1 and TRPC1 at the PM *A* and *B*, images from two different rabbit portal vein VSMCs showing TRPC1 (green) and STIM1 (red) staining. Changes in the relative fluorescence of TRPC1 and STIM1 staining across the cell width were determined from the line scan (dotted white line). In a resting cell (*A*), TRPC1 staining was predominantly present at the PM, whereas labelling for STIM1 was located within the cytosol. In a cell treated with BAPTA‐AM for 10 min (*B*), both TRPC1 and STIM1 were located at the PM in discrete puncta. Inset: co‐localization between TRPC1 and STIM1 staining (yellow) at the PM. *C* and *D*, PLA images showing BAPTA‐AM induced fluorescence signals between STIM1 and TRPC1 which were predominantly at the PM in rabbit portal vein and mice mesenteric artery VSMCs. *E*, immunocytochemical image showing that, in TRPC1^−/−^ mesenteric artery VSMCs, BAPTA‐AM induced translocation of STIM1 from the cytosol to the PM where it produced uniform, non‐puncta‐like staining.

In freshly isolated unstimulated VSMCs, immunocytochemical studies showed that STIM1 staining (red) was mainly located within the cytosol, TRPC1 staining (green) was predominantly found at the PM, and there were sparse apparent regions of co‐localization (Fig. 6 *A*). Pre‐treatment with BAPTA‐AM caused translocation of STIM1 signals from the cytosol to the PM, and also stimulated co‐localizations between TRPC1 and STIM1 (yellow) at puncta‐like sites (Fig. 6 *B*). Moreover, PLA studies showed no apparent signals between STIM1 and TRPC1 in unstimulated VSMCs, whereas robust fluorescence signals (red) were found at the PM following pre‐treatment with BAPTA‐AM (Fig. 6 *C* and *D*). These findings clearly indicate that store depletion stimulates formation of STIM1‐TRPC1 complexes at the PM. Interestingly, in TRPC1^−/−^ VSMCs, BAPTA‐AM caused the translocation of STIM1 signals to the PM, which had a relatively even distribution without obvious discrete puncta‐like formation (Fig. 6 *E*).

**Figure 7:**
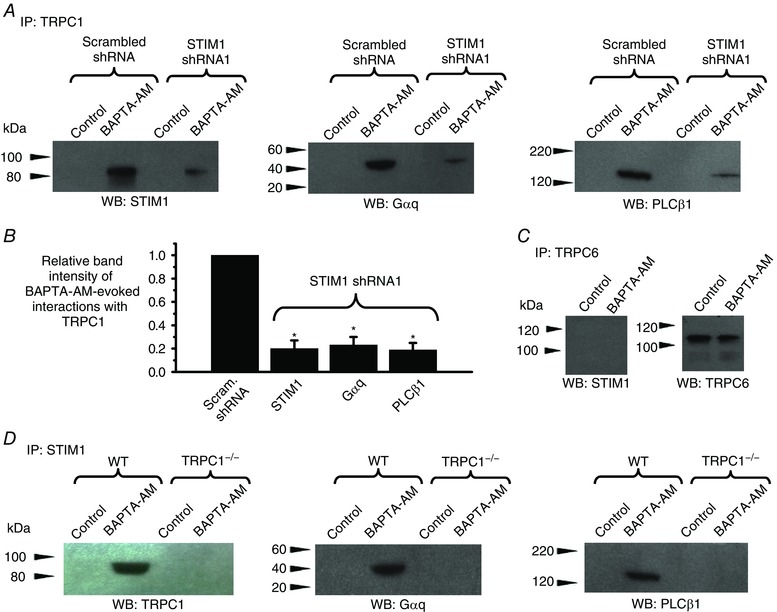
Store‐depletion evokes associations between TRPC1, STIM1, Gαq and PLCβ1 *A*, western blots showing that pre‐treatment with BAPTA‐AM for 10 min induced associations between TRPC1 and STIM1, Gαq and PLCβ1, which were reduced by STIM1 shRNA1. Primary cultured rabbit portal vein VSMC lysates were initially immunoprecipitated (IP) with anti‐TRPC1 antibodies and were then western blotted (WB) with anti‐STIM1, anti‐Gαq or anti‐PLCβ antibodies. *B*, mean data for relative band intensities of BAPTA‐AM‐evoked interactions with TRPC1 shown in *A* (three different cell lysates; ^*^ *P *< 0.05). *C*, application of BAPTA‐AM for 10 min did not alter interactions between TRPC6 and STIM1 (left) or change expression levels of TRPC6 (right) in rabbit portal vein tissue lysates. *D*, primary cultured WT mesenteric artery VSMCs: BAPTA‐AM evoked interactions between STIM1 and TRPC1, Gαq and PLCβ1, which were absent in cell lysates from TRPC1^−/−^ VSMCs.

**Figure 8:**
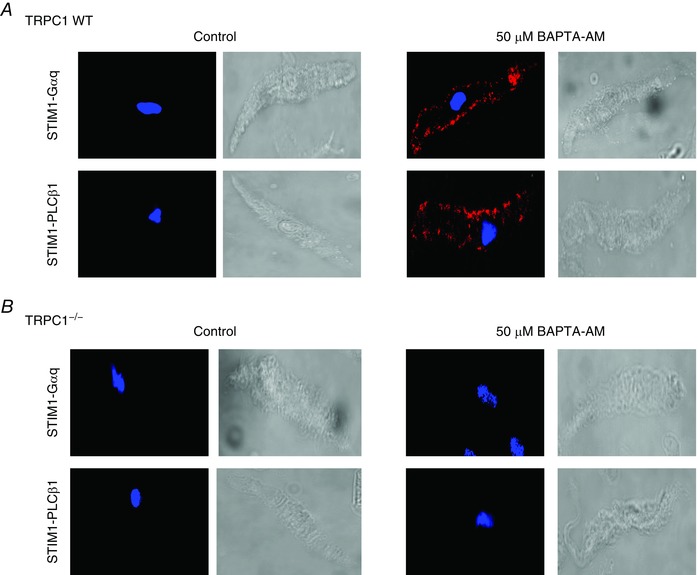
Store‐operated induced interactions between STIM1, Gαq and PLCβ1 require TRPC1 *A*, PLA images from WT mesenteric artery VSMCs showing that BAPTA‐AM induced interactions between STIM and Gαq, and also between STIM1 and PLCβ1. *B*, BAPTA‐AM‐evoked interactions between STIM and Gαq, and STIM1 and PLCβ1 were absent in TRPC1^−/−^ VSMCs.

Because Gαq/PLCβ1 activity is obligatory for TRPC1 channel activation in VSMCs (Shi *et al*. 2016), we next proposed that store‐operated STIM1‐TRPC1 complexes probably also encompass Gαq and PLCβ1. In unstimulated primary cultured VSMCs expressing scrambled shRNA, co‐immunoprecipitation studies showed that TRPC1 did not associate with STIM1, Gαq or PLCβ1 (Fig. 7 *A*). However, pre‐treatment with BAPTA‐AM induced significant interactions between these molecules (Fig. 7 *A*). Similar interactions between TRPC1 and STIM1 were also obtained with CPA (data not shown). Knockdown of STIM1 with STIM1 shRNA1 significantly decreased BAPTA‐AM‐stimulated associations between TRPC1 and STIM1, Gαq and PLCβ1 (Fig. 7 *A* and *B*). Correspondingly, BAPTA‐AM‐activated interactions between STIM1 and TRPC1, Gαq and PLCβ1 measured using co‐immunoprecipitation (Fig. 7 *D*) and PLA (Fig. 8) were present in WT but absent in TRPC1^−/−^ cell lysates.

In control experiments, BAPTA‐AM and CPA did not alter expression levels of TRPC1, STIM1, Gαq and PLCβ1 (Shi *et al*. 2016), TRPC1 expression was not altered in the presence of shRNA STIM1, and STIM1 expression was not changed in WT and TRPC1^−/−^ vessel lysates (data not shown).

TRPC6 subunits form receptor‐operated Ca^2+^‐permeable cation channels in VSMCs that are not activated by store depletion (Abramowitz & Birnbaumer, 2009; Large *et al*. 2009; Albert, 2011; Earley & Brayden, 2015). In accordance with these findings, we found that TRPC6 proteins were not associated with STIM1 at rest or following pre‐treatment with BAPTA‐AM in vessel lysates (Fig. 7 *C*).

**Figure 9:**
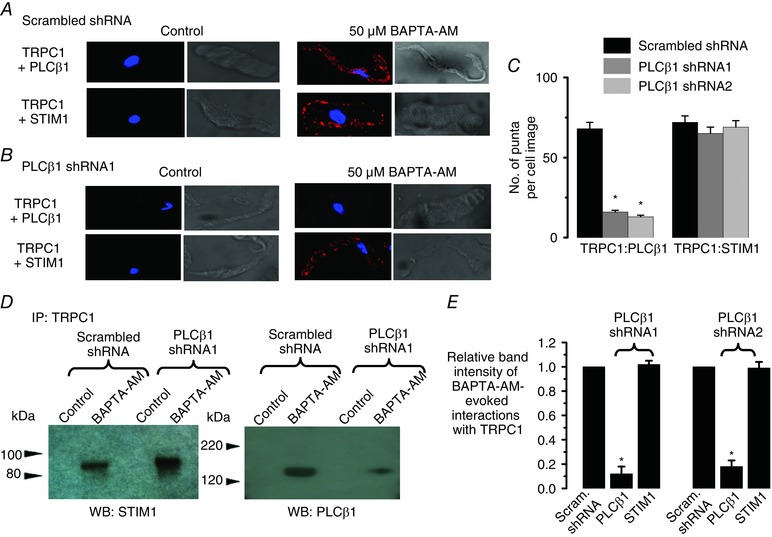
Store‐operated interactions between TRPC1 and STIM1 do not require PLCβ1 *A*–*C*, application of BAPTA‐AM for 10 min induced interactions between TRPC1 and PLCβ1 in rabbit portal vein VSMCs measured using PLA that were reduced by expression of PLCβ1 shRNA1 and shRNA2 sequences, whereas associations between TRPC1 and STIM1 were unaffected (three different preparations; ^*^ *P *< 0.05). *D* and *E*, BAPTA‐AM‐induced interactions between TRPC1 and PLCβ1 measured using co‐immunoprecipitation were reduced by expression of PLCβ1 shRNA1 and shRNA2 sequences, whereas associations between TRPC1 and STIM1 were unaffected (three different rabbit portal vein cell lysates; ^*^ *P *< 0.05).

Taken together, these results suggest that store‐operated formation of STIM1‐TRPC1 complexes is required before interactions with Gαq and PLCβ1 occur. To further explore this idea, we studied the effect of decreasing expression of PLCβ1 on store‐operated interactions between TRPC1 and STIM1. In VSMCs expressing scrambled shRNA, PLA studies showed that BAPTA‐AM induced interactions between TRPC1 and PLCβ1, and STIM1 (Fig. 9A and *C*). However, in the presence of PLCβ1 shRNA1 and 2, BAPTA‐AM‐evoked interactions between TRPC1 and PLCβ1 were greatly reduced, whereas association between TRPC1 and STIM1 remained unaffected (Fig. 9 *B* and *C*). Similar results following expression of PLCβ1 shRNA1 and 2 were obtained for BAPTA‐AM‐induced interactions between TRPC1 and PLCβ1, and STIM1 using co‐immunoprecipitation (Fig. 9 *D* and *E*). In control experiments, PLCβ1 shRNA1 and 2 did not alter expression of TRPC1, Gαq and STIM1 (data not shown).

### STIM1 acts as a cell surface transmembrane protein to activate TRPC1‐based SOCs

In the present study, we have shown that application of the N‐terminal EF hand STIM1 antibody to the extracellular surface of VSMCs inhibited store‐operated TRPC1‐based SOCs (Fig. 2 *B*). This raises the possibility that store depletion may induce translocation of STIM1 from the intracellular compartment to the PM where it acts a transmembrane protein to interact with TRPC1 and stimulate PLCβ1 activity. To further investigate this idea, we compared the effect of the N‐terminal STIM1 antibody with an antibody raised against a C‐terminal region of STIM1 (Prakriya & Lewis, 2015) on activation of TRPC1‐based SOCs and on store‐operated translocation of STIM1 from the cytoplasm to the PM.

**Figure 10:**
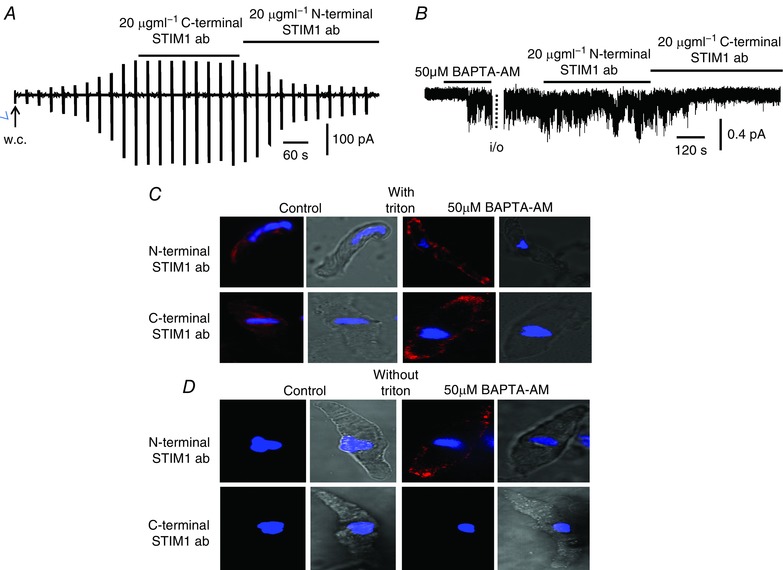
Differential actions of antibodies raised against N‐terminal and C‐terminal regions of STIM1 on activation of TRPC1‐based SOCs *A*, original trace showing that a store‐operated whole‐cell current from a WT mesenteric artery VSMC was inhibited by bath application of a N‐terminal but not a C‐terminal STIM1 antibody. *B*, representative recording showing that BAPTA‐AM‐evoked single channel activity in an inside‐out patch from a WT VSMC held at −80 mV was inhibited by bath application of a C‐terminal but not a N‐terminal STIM1 antibody. *C*, representative images from two different VSMCs treated with Triton, in which both N‐terminal and C‐terminal STIM1 antibodies WT identified translocation of STIM1 signalling (red) from the cytosol to the PM following treatment with BAPTA‐AM for 10 min. *D*, representative images from two different WT VSMCs not treated with Triton, in which N‐terminal, nor C‐terminal STIM1 antibodies identified STIM1 staining in unstimulated cells, and only the N‐terminal antibody revealed STIM1 staining at the PM following treatment with BAPTA‐AM.

In freshly isolated WT VSMCs, bath application of the N‐terminal STIM1 antibody greatly reduced the mean amplitude of store‐operated whole‐cell currents from 4.6 ± 0.8 pA/pF to 1.6 ± 0.4 pA/pF (*n* = 6) at −80 mV but had little effect on BAPTA‐AM‐evoked single‐channel activity when bath applied to the cytosolic surface of inside‐out patches (Fig. 10 *A* and *B*). By contrast, bath application of the C‐terminal STIM1 antibody had little effect on store‐operated whole‐cell currents but produced pronounced inhibition of the mean NP_o_ value of BAPTA‐AM‐evoked single‐channel activity from 0.63 ± 0.05 to 0.13 ± 0.02 (*n* = 6) at −80 mV when applied to the cytosolic surface of inside‐out patches (Fig. 10 *A* and *B*). Immunocytochemical studies also revealed differential effects of N‐terminal and C‐terminal STIM1 antibodies on STIM1 staining according to whether cells were permeabilized with Triton. In unstimulated VSMCs, N‐terminal and C‐terminal antibodies only produced staining for STIM1 when cells were treated with Triton (Fig. 10 *C* and *D*). However, BAPTA‐AM‐evoked translocation of STIM1 signals to the PM were recorded using the N‐terminal STIM1 antibody in both Triton and non‐Triton treated VSMCs, whereas BAPTA‐AM‐evoked STIM1 translocation to the PM was only recorded with the C‐terminal STIM1 antibody in cells pre‐treated with Triton (Fig. 10 *C* and *D*).

These findings further indicate that, in resting cells, STIM1 is probably found within the cell not the PM. Furthermore, activation of TRPC1‐based SOCs by STIM1 may involve store‐operated STIM1 translocation to the PM where it acts as a transmembrane protein, with its N‐terminal region exposed to the extracellular environment and its C‐terminal remaining within the cytosol. It is perhaps in this configuration that TRPC1‐STIM1 interactions stimulate PLC activity.

### Noradrenaline‐evoked TRPC1 activation requires STIM1

**Figure 11:**
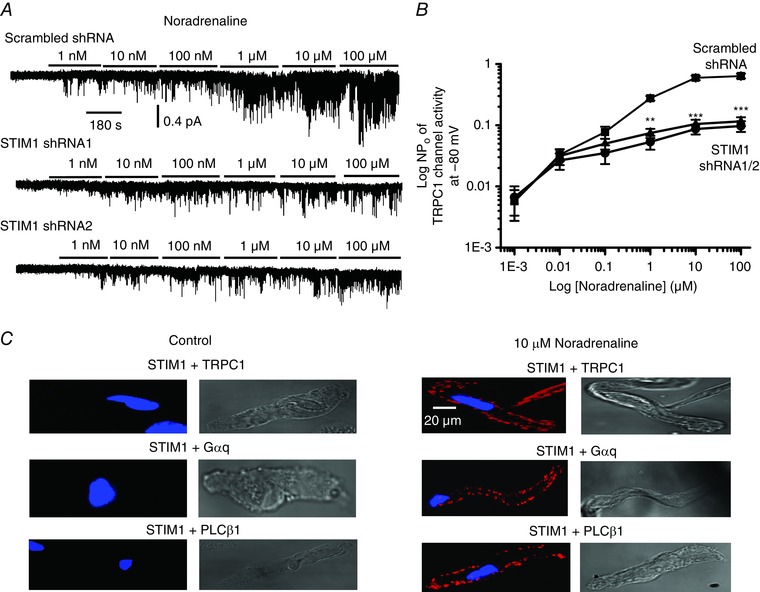
Noradrenaline‐evoked TRPC1 channel activity is mediated by STIM1 *A*, bath application of noradrenaline evoked TRPC1 channel activity in a concentration‐dependent manner in cell‐attached patches from WT mesenteric artery VSMCs held at −80 mV, which were reduced in VSMCs expressing STIM1 shRNA1 and shRNA2 sequences compared to scrambled shRNA. *B*, mean data showing the inhibitory actions of STIM1 shRNA1 and shRNA2 on noradrenaline‐evoked TRPC1 channel activity (at least six patches in which every concentration of noradrenaline tested was cumulatively applied; ^**^ *P* < 0.01, ^***^ *P* < 0.001). *C*, PLA images from single WT VSMCs showing that application of noradrenaline for 5 min induced fluorescence signals (red), which indicated interactions between TRPC1 and STIM1, STIM1 and Gαq, and STIM1 and PLCβ1 predominantly at the PM.

The results of the present study clearly demonstrate that STIM1 is critical for stimulation of native TRPC1 channels by agents that deplete internal Ca^2+^ stores. In a final series of experiments, we investigated whether STIM1 is involved in activation of TRPC1 channels by the vasoconstrictor noradrenaline. In WT VSMCs expressing scrambled shRNA, bath application of noradrenaline activated 2 pS TRPC1 channel activity in cell‐attached patches held at −80 mV in a concentration‐dependent manner as described previously (Shi *et al*. 2016). In the presence of STIM1 shRNAs, stimulation of TRPC1 channel activities by concentrations of noradrenaline >1 μm were reduced by >80% (Fig. 11 *A* and *B*). In PLA experiments, noradrenaline induced interactions between STIM1 and TRPC1, Gαq and PLCβ1 (Fig. 11 *C*). These results strongly suggest that STIM1 is required for activation of native TRPC1 channel activity by noradrenaline.

## Discussion

The present study reveals that, in contractile VSMCs, depletion of intracellular Ca^2+^ stores causes STIM1 to translocate from within the cell to the PM where it interacts with TRPC1 to induce channel opening through stimulation of Gαq‐evoked PLCβ1 activity. In other cell types, STIM1 has been proposed to combine directly with TRPC‐based SOCs and Orai1‐based CRACs to cause channel opening (Worley *et al*. 2007; Yuan *et al*. 2009; Lee *et al*. 2014; Asannov *et al*. 2015; Prakriya & Lewis, 2015) and therefore these findings represent a novel activation pathway of TRPC1‐based SOCs and a previously unrecognized role for STIM1.

### TRPC1‐based SOCs require STIM1 for activation in contractile VSMCs

We show that several well‐established store depletion protocols activate whole‐cell conductances with a relatively linear *I*/*V* relationship and an *E*_rev_ of +20 mV and single‐channel currents with a unitary conductance of ∼2 pS, which are inhibited by TRPC1 antibodies and absent in TRPC1^−/−^ VSMCs. Knockdown of STIM1 using two STIM1 shRNA sequences and two antibodies raised against N‐ and C‐terminal regions of ER/SR Ca^2+^ store sensor STIM1 also produced pronounced inhibition of these store‐operated whole‐cell and single‐channel currents. These antibodies probably have a selective action because they identify a protein band with a molecular weight corresponding to STIM1, which has a reduced density in the presence of shRNA STIM1. In addition, the N‐terminal STIM1 antibody has been shown to reduce STIM1‐evoked *I*_crac_ (Spassova *et al*. 2006). Knockdown of STIM1 also reduced store‐operated PKC phosphorylation of TRPC1, which is a known pivotal event for activation of TRPC1‐based SOCs in contractile VSMCs (Saleh *et al*. 2008; Albert *et al*. 2009; Large *et al*. 2009; Albert 2011; Shi *et al*. 2012). These findings confirm earlier reports that native TRPC1‐based SOCs are expressed in contractile VSMCs (Saleh *et al*. 2008; Albert *et al*. 2009; Large *et al*. 2009; Albert 2011; Shi *et al*. 2012) and highlight, for the first time, that STIM1 is an obligatory molecule in the PKC‐mediated gating pathway of these channels.

### Store‐operated STIM‐TRPC1 interactions stimulate PLCβ1 activity

There is substantial evidence that PKC activity is essential for stimulation of TRPC1‐based SOCs in contractile VSMCs (Saleh *et al*. 2008; Albert *et al*. 2009; Large *et al*. 2009; Albert 2011; Shi *et al*. 2012) and, in a recent study, we reported that store depletion stimulates Gαq‐evoked PLCβ1 activity to drive PKC activity and channel opening (Shi *et al*. 2016). We therefore hypothesized that, because STIM1 is an ER/SR Ca^2+^ sensor, its role in activating TRPC1‐based SOCs may be through coupling store depletion to stimulation of Gαq/PLCβ1 activity.

Store‐operated PLC activity recorded using the PIP_2_/InsP_3_ biosensor GFP‐PLCδ1‐PH (Balla & Vamai, 2009; Szentpetery *et al*. 2009; Shi *et al*. 2016) was inhibited by knockdown of STIM1, and was absent in TRPC1^−/−^ VSMCs, which indicates that both STIM1 and TRPC1 are required to stimulate PLC activity. Immunocytochemical and PLA studies identified that store depletion stimulated the translocation of STIM1 from within the cell to the PM, where it formed STIM1‐TRPC1 complexes with puncta‐like distributions. The discrete pattern of STIM1‐TRPC1 complex distribution appeared to be dependent on TRPC1 because relatively even, non‐puncta‐like STIM1 staining was produced by store depletion in TRPC1^−/−^ cells. In further agreement with STIM1‐TRPC1 complexes stimulating Gαq/PLCβ1 activity, co‐immunoprecipitation studies revealed that store depletion activate associations between TRPC1 and Gαq, and PLCβ1, as well as between STIM1 and Gαq, and PLCβ1, with these interactions requiring both STIM1 and TRPC1. Knockdown of PLCβ1 did not affect formation of store‐operated STIM1‐TRPC1 interactions, which further implies that STIM1‐TRPC1 interactions occur before Gαq/PLCβ1 binding. It is not yet known whether store‐operated STIM1‐TRPC1 interactions require Gαq subunits, and whether association and stimulation of Gαq and PLCβ1 activities occur with STIM1‐TRPC1 interactions in a sequential pathway.

Store depletion failed to induce interactions between STIM1 and TRPC6, which forms non‐TRPC1 containing receptor‐operated Ca^2+^‐permeable channels in VSMCs that are not activated by store depletion (Large *et al*. 2009; Albert, 2011). This agrees with over‐expression studies showing that store depletion activates interactions between STIM1 and TRPC1‐, TRPC4‐ and TRPC5‐based SOCs but does not stimulate interactions between STIM1 and TRPC3, TRPC6 and TRPC7 that are not activated by store depletion (Worley *et al*. 2007). Thus, interactions with STIM1 are probably key in determining whether TRPC channels are activated by store depletion.

STIM1 has diverse cellular partners including ion channels such as Orai1 channels (Prakriya & Lewis, 2015), TRPC channels (Cheng *et al*. 2013), voltage‐gated Ca^2+^ channels (Park *et al*. 2010; Wang *et al*. 2010), SR and PM Ca^2+^‐ATPases (Jousset *et al*. 2007; Ritchie *et al*. 2012), and adenylyl cyclases (Lefkimmiatis *et al*. 2009). The present study makes an important addition to this list: store‐operated formation of STIM1‐TRPC1 complexes that stimulate Gαq/PLCβ1 activity.

### STIM1 acts at the PM to activate TRPC1 channels

It is not clear how store‐operated STIM1‐TRPC1 interactions induce Gαq/PLCβ1 activity. Over‐expression studies generally indicate that activation of Orai1‐based CRACs and TRPC‐based SOCs by STIM1 involves movement of the ER membrane containing activated STIM1 towards the PM, where junction‐like complexes are formed and STIM1 binds to intracellular domains of channel proteins to assemble Orai1‐based CRACs by protein–protein interactions (Prakriya & Lewis, 2015) and directly gate TRPC1‐based SOCs using electrostatic and protein–protein interactions (Worley *et al*. 2007; Yuan *et al*. 2009; Lee *et al*. 2014; Asannov *et al*. 2015). Although electrostatic gating is unique to TRPC1, protein–protein interactions between STIM1 and CRAC and STIM1 and TRPC1 channels involve the same 100 amino acid stretch of STIM1 referred to as SOAR or CAD (Asannov *et al*. 2015). STIM1 has also been suggested to act at, or within, the PM to activate Orai1‐based CRACs (Zhang *et al*. 2005; Spassova *et al*. 2006; Hewavitharana *et al*. 2008) and constitutively active STIM1 present in the PM is proposed to be required for activation of arachidonic acid‐regulated Ca^2+^ channels composed of Orai1 and Orai3 subunits (Thomson & Shuttleworth, 2013).

Our findings of differential inhibitory actions of N‐ and C‐terminal STIM1 antibodies on activation of TRPC1‐based SOCs suggest that, in contractile VSMCs, store‐operated STIM1 is translocated from the SR into the PM. Here, it probably continues to act as a transmembrane protein through its proposed transmembrane domain located between amino acids 214 and 234 (Soboloff *et al*. 2012), with its N‐terminal region presented to the extracellular environment and its C‐terminal region remaining within the cell. It is in this configuration that STIM1 probably forms complexes with TRPC1 that allow subsequent binding and stimulation of Gαq/PLCβ1 activity. These conclusions indicate that STIM1 involved in interacting with TRPC1 and stimulating Gαq/PLCβ1 activity is derived from within the cell, and is not part of STIM1 pools constitutively present in the PM. Future experiments are needed to examinine whether previously identified interactions sites between STIM1 and TRPC1 (Worley *et al*. 2007; Yuan *et al*. 2009; Lee *et al*. 2014; Asannov *et al*. 2015) are involved in association with Gαq and PLCβ1. Understanding the associations between STIM1‐TRPC1 and Gαq subunits are particularly important because these are probably the trigger for initiating PLCβ1 activity that drives channel gating.

### Involvement of Orai proteins involved in the composition and activation of TRPC1‐based SOCs in contractile VSMCs

There is debate concerning whether functional TRPC‐based SOCs require involvement of Orai proteins, which may act as pore‐forming subunits or obligatory accessory proteins, or may mediate Ca^2+^ signals that regulate TRPC expression at the PM (Cheng *et al*. 2011; Cheng *et al*. 2013; Liao *et al*. 2014; Prakriya & Lewis, 2015). In synthetic, non‐contractile VSMCs, store depletion stimulates Ca^2+^ entry involving both TRPC1 and Orai1, and also activates a conductance with *I*_crac_‐like properties (Li *et al*. 2008; Ng et al., 2009, 2010; Li *et al*. 2011; Beech, 2012; Trebak, 2012). In contractile VSMCs, the present study failed to detect store‐operated conductances with characteristics of Orai1‐, Orai2‐ and Orai3‐based SOCs such as strong inward rectification and an *E*_rev_ >+50 mV (Lis *et al*. 2007; Prakriya & Lewis, 2015) in TRPC1^−/−^ VSMCs, and in WT cells when TRPC1‐based SOCs were inhibited with a TRPC1 antibody. This indicates that Orai proteins probably do not contribute to activation of TRPC1‐based SOCs in contractile VSMCs, which is in agreement with a low level of Orai protein expression in these cells (Berra‐Romani *et al*. 2008; Trebak, 2012). In a wider context, this present study provides further evidence that cells express multiple SOCs composed of TRPC‐based SOCs and Orai‐based CRACs, which can exist independently of one another.

It is possible that Orai‐based CRACs are present in contractile VSMCs but that opening of these channels produces small irresolvable conductances using electrophysiological techniques. An alternative approach may be to investigate whether Orai‐based Ca^2+^ sparklets are present using total internal reflection fluorescence microscopy, which can detect Ca^2+^ entry through opening of Ca^2+^‐permeable channels at localized regions of the PM (Sonkusare *et al*. 2012). It is clear that a detailed comparison on the role of Orai proteins in activation of native TRPC1‐based SOCs in contractile and synthetic VSMCs is needed.

### Physiological importance of STIM1‐activated TRPC1 channels

TRPC1 channel activity induced by the endogenous α1 adrenergic Gαq‐coupled receptor agonist and vasoconstrictor noradrenaline was prevented by knockdown of STIM1. Noradrenaline also evoked interactions between TRPC1 and STIM1, STIM1 and Gαq, and STIM1 and PLCβ1 at the PM. These results indicate that STIM1 is important for activation of TRPC1 channels by a physiologically agonist, and suggests that this may involve a similar activation pathway induced by agents that deplete Ca^2+^ stores. This is further highlighted by previous data showing that noradrenaline‐evoked TRPC1 channel activity is also inhibited by knockdown of PLCβ1 (Shi *et al*. 2016).

Physiological vasoconstrictors acting through Gαq‐coupled receptors stimulate PLC, leading to InsP_3_‐mediated depletion of Ca^2+^ stores and DAG‐mediated PKC activation. It therefore might be expected that the store‐operated STIM1/PLCβ1/PKC pathway described in the present study represents a feed forward mechanism to induce TRPC1 channel opening. However, our data indicate that STIM1 and PLCβ1 contribute little to overall PLC activation by noradrenaline (Figs 4 *B* and *C* and 5 *B*) (Shi *et al*. 2016). This may suggest that the STIM1/PLCβ1/PKC pathway involved in activating TRPC1 channels is uniquely stimulated following store depletion. To confirm these ideas, it will be important to determine which PLC isoform is coupled to stimulation of α1 Gαq‐coupled adrenoreceptors, and also to identify whether Gαq receptor/PLC‐coupled DAG generation activates a PKC isoform different from that induced by store‐operated PLCβ1 activity. Moreover, these ideas should be tested using different vasoconstrictor agents such as angiotensin II and endothelin‐1, which are known to stimulate TRPC1‐based SOCs (Albert *et al*. 2009; Large *et al*. 2009).

### Conclusions

**Figure 12:**
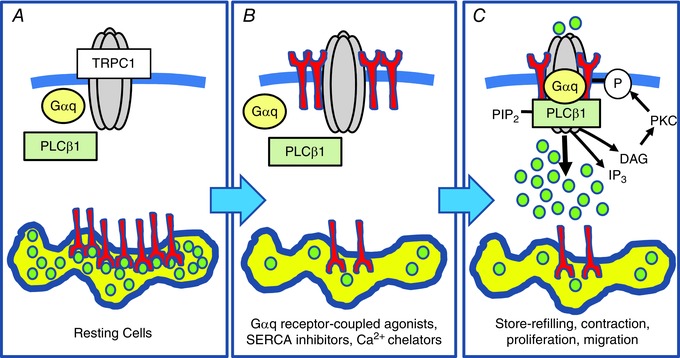
Proposed activation model of TRPC1‐based SOCs in contractile VSMCs *A*, in resting VSMCs SR stores are filled with Ca^2+^, TRPC1‐based SOCs do not interact with Gαq, PLCβ1 or STIM1, and the channels are in a closed state. *B*, following store depletion of the SR, STIM1 proteins (red) are activated and translocated from the SR into the PM, where they interact with TRPC1. Following translocation, the N‐terminal EF hand of STIM1, which acts as a Ca^2+^ sensor within the SR, is exposed on the external surface of the cell, whereas the C‐terminal region is maintained within the cytosol. *C*, formation of store‐operated STIM1‐TRPC1 interactions enable the binding of Gαq and PLCβ1, which stimulate PLC activity, leading to PIP_2_ hydrolysis, formation of DAG, PKC stimulation, phosphorylation of TRPC1 subunits and channel opening.

The present study describes a novel activation pathway of TRPC1‐based SOCs in native contractile VSMCs (Fig. 12). Store depletion stimulates translocation of STIM1 from the SR to the PM where it is inserted as a transmembrane protein and forms STIM1‐TRPC1 complexes, which subsequently bind and stimulation of Gαq/PLCβ1 activities. This increase in PLCβ1 activity stimulates channel opening through DAG‐evoked PKC phosphorylation of TRPC1.

## Additional information

### Competing interests

The authors declare that they have no competing interests.

### Author contributions

APA, WAL, LB, JS and FM contributed to the conception or design of the work, analysis of data or interpretation of data for the work, and were involved in drafting the work or revising it critically for important intellectual content. JS was involved in the acquisition of data. All authors approved final version of the manuscript and agree to be accountable for all aspects of the work. All persons designated as authors qualify for authorship, and all those who qualify for authorship are listed.

### Funding

This work was supported by the Biotechnology and Biological Sciences Research Council (BB/J007226/1 and BB/M018350/1 to APA) and was also supported in part by the NIH Intramural Research Program (Project Z01‐ES‐101684 to LB).
